# Calcified vs. Non-Calcified Tendinopathy of the Rotator Cuff: Clinical Presentations, Prognostic Implications, and Emerging Therapeutic Strategies

**DOI:** 10.26502/josm.511500218

**Published:** 2025-08-04

**Authors:** Hina P. Patel, Shaan Patel, Michael Zalin, Devendra K. Agrawal

**Affiliations:** Department of Translational Research, College of Osteopathic Medicine of the Pacific, Western University of Health Sciences, Pomona, California, 91766, USA

**Keywords:** Calcific tendinopathy, Extracorporeal shockwave therapy, Inflammation, Musculoskeletal disease, Non-calcified tendinopathy, Rotator cuff injury, Ultrasound-guided barbotage

## Abstract

Rotator cuff tendinopathy is a common cause of shoulder pain and dysfunction, presenting in two primary forms: calcific and non-calcific. These subtypes differ significantly in their pathophysiology, clinical manifestations, and natural history, necessitating tailored diagnostic and therapeutic approaches. This review delineates the clinical presentations of calcific rotator cuff tendinopathy (RCCT), characterized by distinct pre-calcific, calcific, and post-calcific stages, and contrasts them with the more insidious, degenerative course of non-calcific rotator cuff tendinopathy. Diagnostic imaging, particularly musculoskeletal ultrasound, plays a pivotal role in differentiating these conditions, offering advantages in cost, accessibility, and dynamic, real-time assessment over MRI. Treatment strategies range from conservative management with NSAIDs and physical therapy to interventional techniques including ultrasound-guided barbotage, extracorporeal shockwave therapy, corticosteroid injections, and emerging regenerative therapies such as platelet-rich plasma and prolotherapy. Despite advances, further high-quality studies are needed to optimize individualized care per rotator cuff tendinopathy classification and to clarify long-term outcomes. This review highlights current evidence and clinical decision-making considerations to improve the diagnosis and management of rotator cuff calcific and non-calcific tendinopathies.

## 1. Introduction

Tendinopathy is a broad term defining tendon disorders that result from overuse, degeneration or injury. Among its various forms, calcified and non-calcified rotator cuff tendinopathy (RCT) represent distinct conditions with different pathophysiological mechanisms. Calcific tendinopathy is caused by calcium deposition, most commonly affecting the rotator cuff tendons or subacromial-subdeltoid bursa (SASD) of the shoulder, though it can also affect the common calcaneal and patellar tendons [1,2]. This is associated with symptoms such as acute and severe pain, stiffness, reduced range of motion, and tenderness in the affected region [3]. Non-calcific tendinopathy results from tendon degeneration without calcification that is often driven by repetitive strain, aging, or overuse [4].

Rotator cuff calcific tendinopathy (RCCT) is a leading cause of shoulder pain, affecting 35–45% of individuals with calcific deposits most commonly in the supraspinatus tendon or the subacromial-subdeltoid bursa [5]. However, as many as 20% of cases remain asymptomatic with rotator cuff calcium deposits identified on imaging [6,7]. It most commonly affects individuals between the ages of 30 and 60 years, with additional risk factors including female sex, diabetes mellitus, hyperlipidemia, hypothyroidism, and a possible genetic predisposition [8]. Despite its prevalence, the exact etiology and pathogenesis of RCCT remain unclear, with hypotheses ranging from degenerative changes and mechanical overload to cell-mediated processes and genetic predisposition [9,10]. The condition can be self-limiting but may also progress to chronic, debilitating symptoms if not managed appropriately. Diagnosis relies on clinical evaluation supported by imaging modalities such as ultrasound or MRI, while treatment options encompass approaches from conservative management, such as physiotherapy and anti-inflammatory medications, to image-guided interventions and surgical removal in refractory cases [11,12]. Despite the unknown direct pathogenesis and etiology, understanding RCCT is crucial for clinicians to ensure accurate diagnosis and to tailor treatment strategies that address both symptom relief and long-term tendon health.

Non-calcific RCT is marked by degenerative structural changes within the tendon, including collagen fiber disorganization, thinning of tendon fibers, neovascularization, and mucoid degeneration [13]. These pathological alterations compromise the tendon’s mechanical integrity and load-bearing capacity, frequently resulting in pain, diminished shoulder function, and, in some cases, progression to partial- or full-thickness rotator cuff tears. Risk factors leading to this repetitive microtrauma include both intrinsic variables like age, poor vascularization, diabetes, and smoking, and extrinsic variables such as overuse and trauma [14]. Management on non-calcified rotator cuff tendinopathy is primarily conservative, including activity modification, physical therapy, administering non-steroidal anti-inflammatory drugs (NSAIDs), and corticosteroid injections [15]. For patients with persistent or refractory symptoms unresponsive to non-operative measures, surgical intervention may be warranted. Current research efforts are focused on elucidating the underlying biological mechanisms of tendon degeneration and advancing regenerative treatment strategies aimed at restoring tendon structure and function.

## 2. Mechanisms of Rotator Cuff Tendinopathy

The etiology of RCT involves both intrinsic and extrinsic factors, or a combination of both. Intrinsic factors are associated with tendon degeneration due to natural aging, altered cellular biology due to metabolic diseases or injury, inflammatory response, poor vascularity, mechanical overuse, and genetic predisposition [16–25]. Tendons rely heavily on parallel, densely packed Type I collagen fibers to withstand tensile loads; however, age-related changes, repetitive stress, tissue injury, and clinical conditions can disrupt this organized microarchitecture, weakening the structural integrity of the tendon [26–28]. These degenerative processes may occur on the articular side of the tendon facing the glenohumeral joint or within the tendon itself [29].

One key site of intrinsic degeneration of the rotator cuff is the enthesis, the fibrocartilaginous interface where tendon attaches to bone. This region is especially prone to rotator cuff injury due to its mechanical role in distributing stress between soft tendon and hard bone during shoulder movement [30–36]. Under normal conditions, the enthesis provides flexibility and protects the tendon from fatigue-related damage. With aging, however, this structure undergoes significant deterioration. Type I fibers within the rotator cuff become disorganized, the tendon-bone attachment zone narrows, and extracellular matrix components such as glycosaminoglycans, proteoglycans, and hydrating polysaccharides diminish [14,16,34]. These intrinsic changes, along with a shift toward weaker, irregularly arranged Type III collagen fibers, ultimately compromise mechanical function and increase susceptibility to RCT [14,16]. Rotator cuff tendons exhibit a characteristic stress-strain curve that reflects their biomechanical performance. At rest, collagen fibers are arranged in a crimped configuration. In the toe region, a 2% strain allows these crimps to straighten without causing damage. In the linear region, a 2–4% strain fully aligns the crimped collagen fibers. This results in elastic deformation if the strain is below 4%, but microscopic damage if it exceeds this threshold. Macroscopic failure typically occurs with 8–10% strain, with complete tendon rupture possible beyond 10% [37–45]. In age-related RCT, degenerative changes such as disorganized collagen, reduced elasticity, and increased Type III collagen shift this curve downward and to the left. As a result, tendons absorb less mechanical load and are increasingly susceptible to tendinopathy, microtears, and rupture even at lower strain thresholds [37–45].

**Figure 1:**
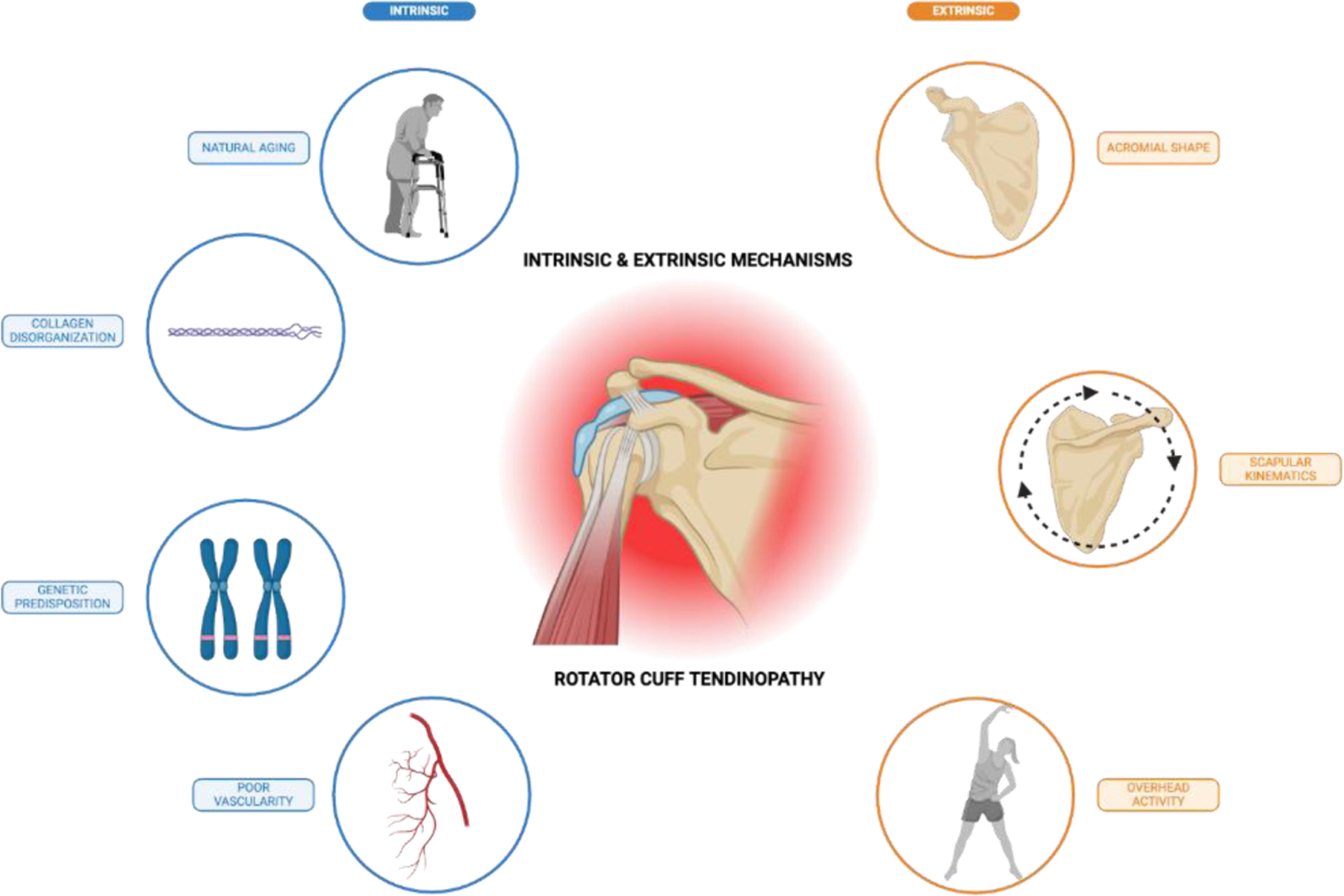
Intrinsic and extrinsic factors contributing to rotator cuff tendinopathy. Rotator cuff tendinopathy arises from a multifactorial interplay of intrinsic and extrinsic factors. Intrinsic mechanisms include natural aging, collagen disorganization, genetic predisposition, and poor vascularity, which compromise tendon structure and function. Extrinsic factors like acromial morphology, altered scapular kinematics, and repetitive overhead activity exert mechanical stress on the rotator cuff tendons, particularly in the subacromial space. Together, these processes contribute to tendon degeneration, impaired healing, and increased susceptibility to both calcific and non-calcific forms of tendinopathy.

Over time, intrinsic degeneration may also lead to narrowing of the subacromial space, resulting in mechanical impingement on the bursal side of the tendon adjacent to the subacromial bursa. Extrinsic anatomical or biomechanical factors, such as acromial shape or altered scapular kinematics, can increase stress on the rotator cuff and compress tendons within a narrowed subacromial space [14,16,17, 44]. Bigliani et al. classified the acromion into three types: flat (Type I), curved (Type II), and hooked (Type III), with the hooked type being strongly associated with impingement and full-thickness tears [46,47]. A flat, horizontally oriented acromion has also been linked to structural degeneration of the rotator cuff, subacromial impingement, and greater loss of function in patients with tendinopathy [14,16,17]. External mechanical compression of the rotator cuff due to bony anatomy alone may not be sufficient to cause RCT, but it can lead to pathological changes when combined with repetitive overhead activity [14,16]. Thus, extrinsic factors may predispose an individual to RCT. In many cases, intrinsic and extrinsic mechanisms coexist and collectively contribute to RCT progression (Figure 1).

## 3. Mechanisms of Rotator Cuff Calcific Versus Non-Calcific Tendinopathy

### 3.1 Rotator Cuff Calcific Tendinopathy

The cumulative degenerative effects of intrinsic and extrinsic mechanisms in RTC may also predispose tendons to calcium hydroxyapatite crystal deposition, resulting in rotator cuff calcific tendinopathy [5]. While the exact pathogenesis of RCCT remains unclear, it is thought to involve primarily cell-mediated processes in which mature tendon cells, or tenocytes, transform into chondrocytes that are responsible for cartilage formation and endochondral ossification [5]. This transformation is likely influenced by intrinsic and extrinsic factors like age-related tendon degeneration, mechanical overload, and inflammation, which disrupt tenocyte homeostasis and activate chondrogenic signaling pathways such as BMPs, Sox9, PTHrp, and FGFR3, priming the tendon environment for pathological calcification.

**Figure 2:**
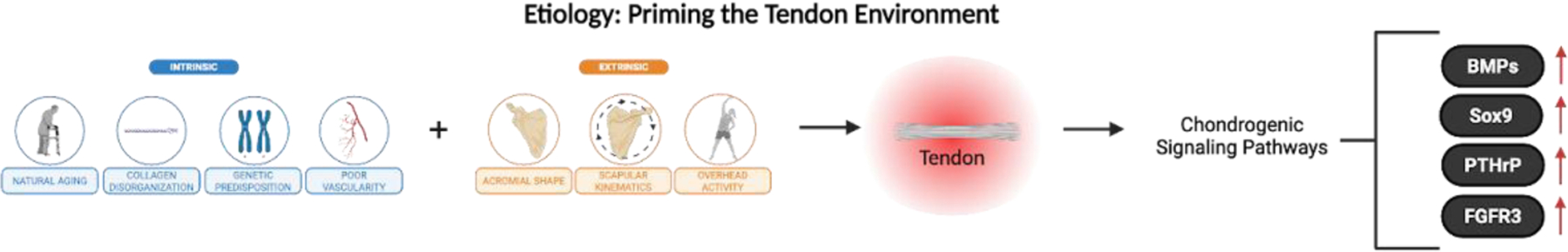
Age-related degeneration, mechanical overload, poor vascularity, and inflammation disrupt tendon homeostasis. These intrinsic and extrinsic factors upregulate key chondrogenic signaling pathways— BMPs, Sox9, PTHrp, and FGFR3—priming tenocytes for pathological transformation.

Histopathological analyses have demonstrated this metaplastic transformation in RCCT, with calcific deposits surrounded by hypertrophic cells exhibiting a chondrocyte-like phenotype and expressing type x collagen [48,49]. Type x collagen, a biomarker for endochondral ossification, is produced by hypertrophic chondrocytes to regulate matrix mineralization and the transition from cartilage to bone [50]. Its presence near calcific deposits within the rotator cuff tendon supports the idea that cell-mediated metaplasia contributes to pathological calcium deposition in RCCT [50]. In addition to type x collagen, Darrieutort-Laffite et al. reported that these chondrocyte-like cells also express tissue non-specific alkaline phosphatase (TNAP) and ectonucleotide pyrophosphatase/phosphodiesterase 1 (ENPP1)—two key enzymes critical to the mineralization process [49]. TNAP degrades pyrophosphate, while ENPP1 hydrolyzes it (Figure 2). Because pyrophosphate inhibits mineralization, TNAP and ENPP1 expression in hypertrophic chondrocytes promotes hydroxyapatite crystal formation and pathological tendon calcification [51,52].

**Figure 3:**
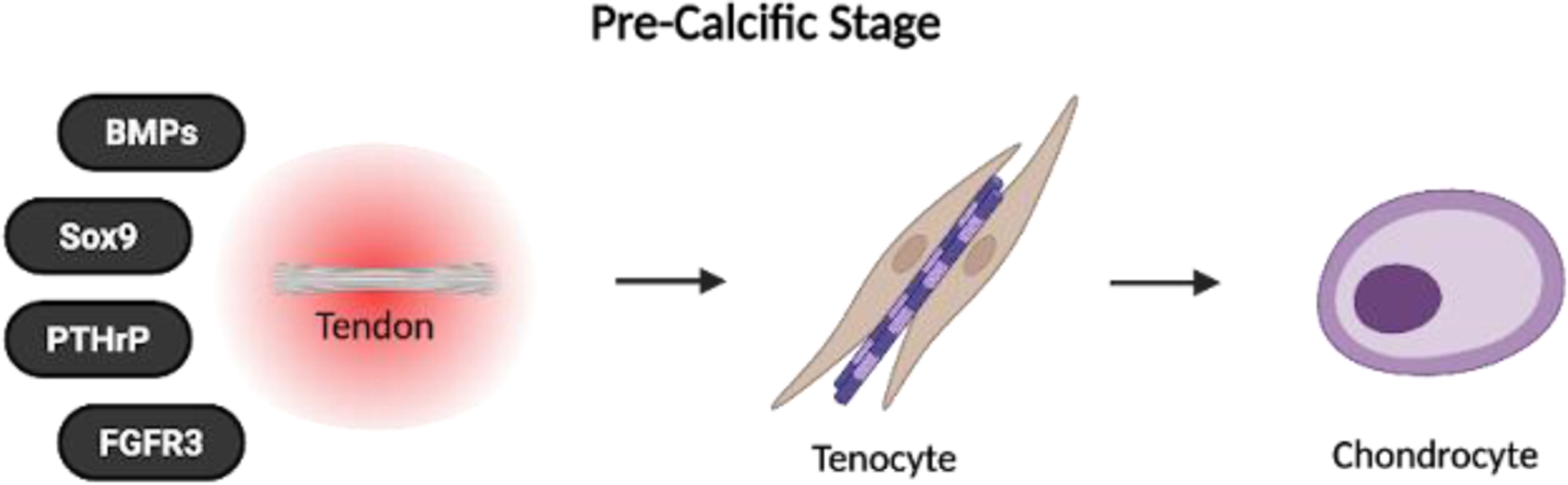
In response to altered signaling, mature tenocytes undergo metaplasia into chondrocyte-like cells. These cells begin to express markers consistent with endochondral ossification.

RCCT is a self-limiting condition that typically resolves over time through a natural sequence of three stages: (i) pre-calcific, (ii) calcific, and (iii) post-calcific [5,51,53]. The pre-calcific stage describes the metaplastic transformation of tenocytes into chondrocyte-like cells that primes the tendon for calcium deposition [5,51,53] (Figure 3).

**Figure 4:**
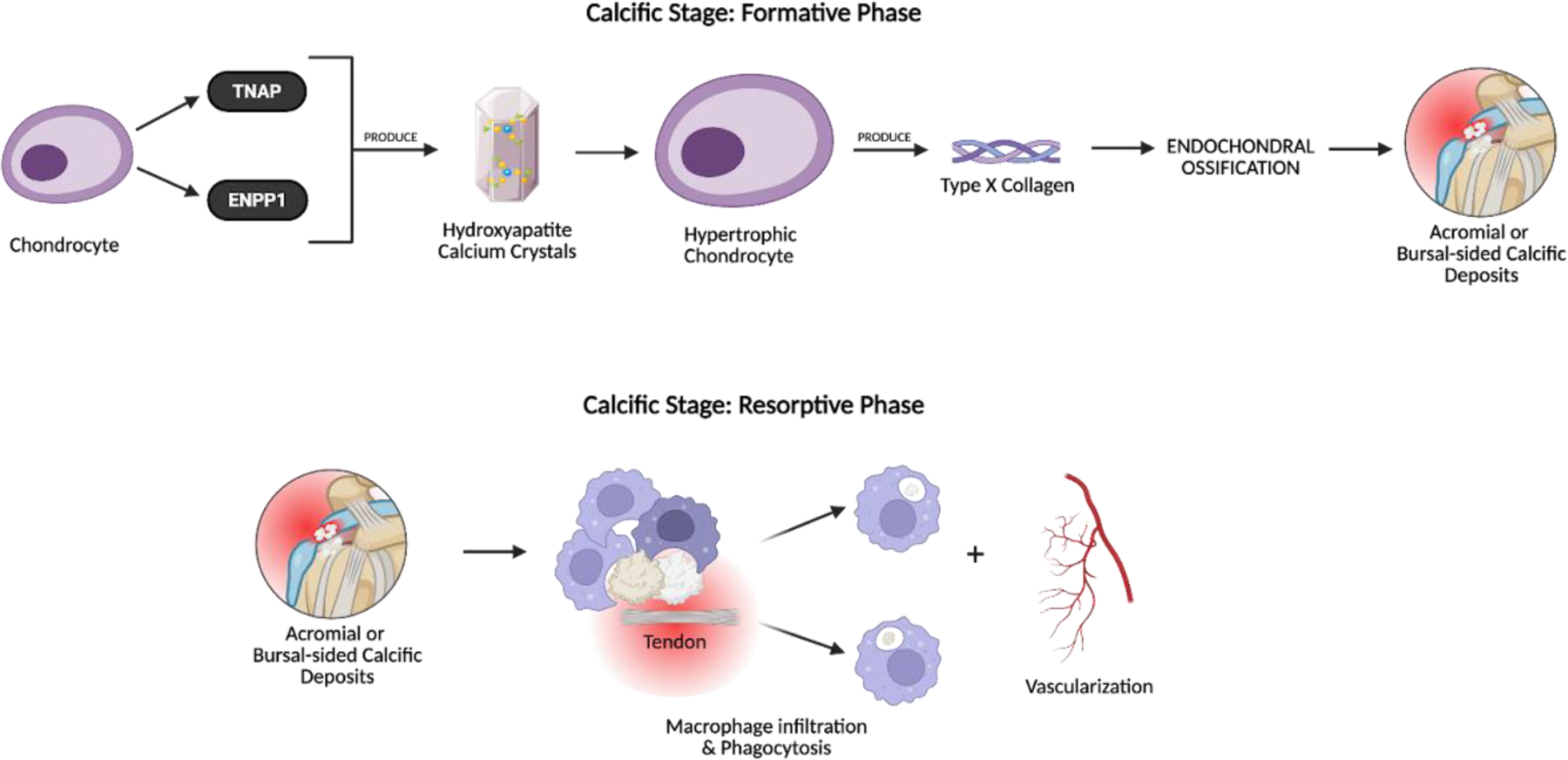
The formative phase includes calcium deposition, in which chondrocyte-like cells express tissue nonspecific alkaline phosphatase (TNAP) and ENPP1, promoting hydroxyapatite crystal deposition within the tendon matrix. Type X collagen production and mineralization follow, initiating the buildup of calcific material. In the resorptive phase, the body initiates a self-limiting immune response. Macrophages infiltrate the tendon to phagocytose calcium and neovascularization accompanies this phase, contributing to significant pain and functional impairment.

The calcific stage is further divided into the formative and resorptive phases. During the formative phase, chondrocytes induce calcium hydroxyapatite crystal accumulation and calcium deposits increase in size [51]. Soon after, the resorptive phase begins and promotes vascularization and macrophage infiltration of the affected area [5,51,53]. The calcium deposits are gradually eliminated via macrophage phagocytosis [3] (Figure 4).

**Figure 5:**
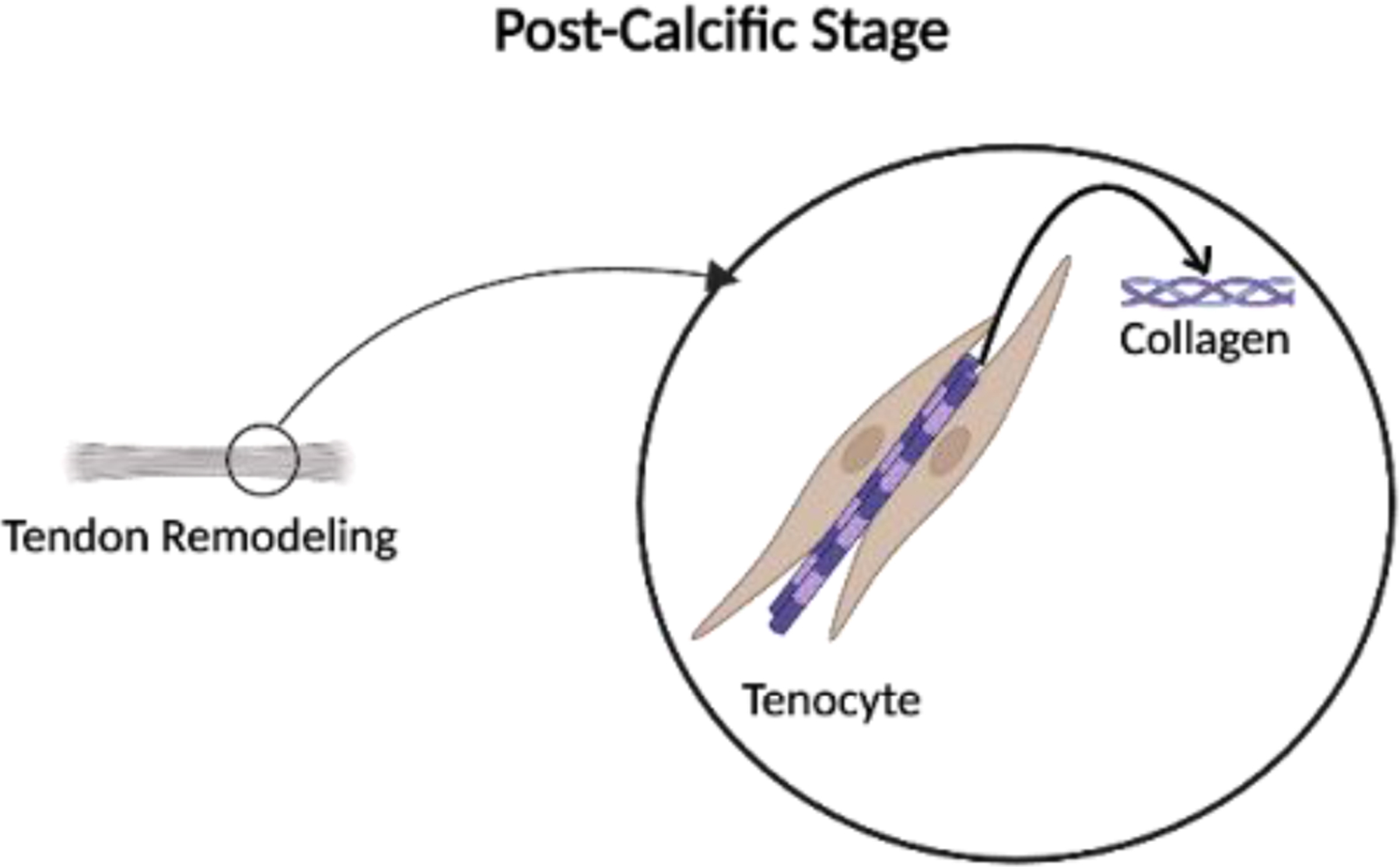
Following the resorptive phase, fibroblasts remodel the extracellular matrix and regenerate normal tendon structure. This phase may overlap with resorption and is associated with symptom resolution and functional recovery.

Lastly, the post-calcific stage overlaps with the resorptive phase and includes fibroblast-mediated tendon remodeling to remove calcium deposits, restore tendon structure, and reduce inflammation [5]. Some patients may recover spontaneously from RCCT, while others might require therapeutic interventions for persisting calcium deposits and severe symptoms (Figure 5).

### 3.2 Rotator Cuff Non-Calcific Tendinopathy

In contrast to the distinct cell-mediated metaplastic and mineralization processes driving calcium deposition in RCCT, intrinsic degenerative changes and extrinsic mechanical overload are central to the progression of non-calcific rotator cuff tendinopathy. As described in Section II, intrinsic factors such as aging, tenocyte senescence, collagen disorganization, and extracellular matrix degradation reduce the tendon’s ability to sustain load and repair microdamage.

Disruption of matrix components like type I collagen and proteoglycans is largely attributed to an upregulation of metalloproteinases (MMPs) [54,55]. In non-calcific RCT, tenocyte inflammation mediated by macrophage infiltration stimulates increased expression of MMPs. Collagenases like MMP-1, MMP-8, and MMP-13 cleave type I collagen, the primary structural protein responsible for the tendon’s tensile strength [54,55]. Reduced amounts of type I collagen and increased prevalence of disorganized type III collagen make the tendon susceptible to injury and degeneration [55].

Extrinsic factors further exacerbate this damage. Hooked acromial morphology, repetitive overhead activity, and scapular dyskinesis contribute to mechanical impingement of the bursal side of the tendon, subjecting it to chronic external compression, friction, and shear [14,16,34]. Additionally, thickening of the coracoacromial ligament may decrease the subacromial space [56]. Factor et al. states that shoulder impingement is the main extrinsic cause of non-calcific RTC [56]. These forces accelerate matrix breakdown and promote a degenerative continuum marked by progression of collagen disorganization, neovascularization, and increased risk of partial- or full-thickness tears [57].

Unlike RCCT, non-calcific RCT lacks a distinct mineralization phase and typically is a chronic, persistent condition that may not resolve without intervention [11]. A deeper understanding of the balance between intrinsic and extrinsic factors in causing non-calcific RCT is critical for guiding tailored therapeutic strategies.

## 4. Clinical Presentations of Rotator Cuff Calcific Versus Non-Calcific Tendinopathy

Rotator cuff calcific and non-calcific tendinopathy differ in their onset, severity, and progression. Recognizing these distinctions is essential for accurate diagnosis and personalized treatment.

### 4.1 Clinical Manifestations of Rotator Cuff Calcific Tendinopathy

The clinical presentation of RCCT depends on whether the patient is in the pre-calcific, calcific, or post-calcific stage [10]. The pre-calcific stage is considered “silent” because the metaplastic transformation of tenocytes into chondrocyte-like cells occurs without inflammation. As a result, patients do not feel pain and are typically asymptomatic due to the absence of calcification or mechanical irritation [10].

**Figure 6:**
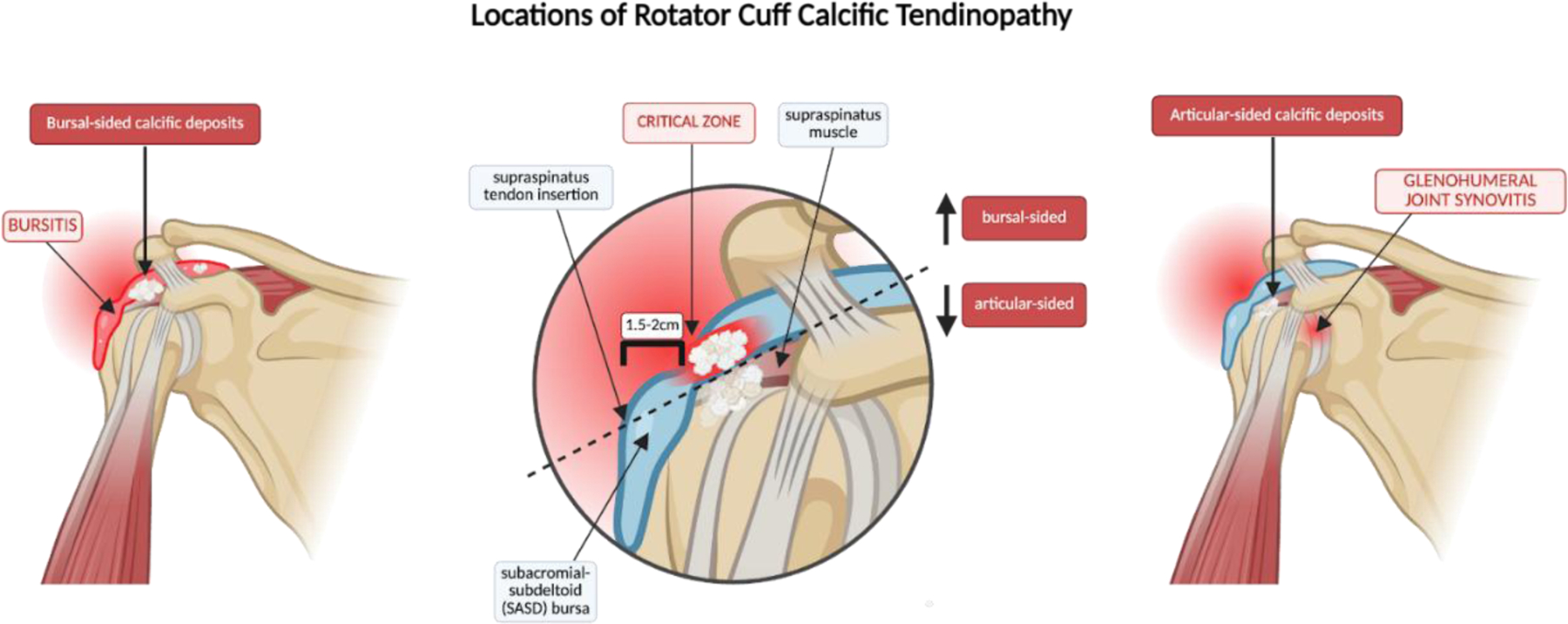
Anatomical localization of calcific deposits in rotator cuff calcific tendinopathy (RCCT). Calcium deposits commonly affect the supraspinatus within the “critical zone”, approximately 1.5–2 cm from its insertion at the greater tubercle of the humerus. These deposits localize to either the articular or bursal side of the tendon, causing symptoms associated with glenohumeral synovitis and subacromial-subdeltoid bursitis, respectively. The dashed line distribution illustrates how localization can influence clinical manifestations and guide interventions.

The calcific stage is associated with “impingement” because calcium hydroxyapatite deposits within the tendon may cause mechanical compression of the adjacent structures, particularly within the subacromial space. Calcific deposits in RCCT can localize to either the articular or bursal side of the tendon, with bursal-sided calcifications more frequently associated with impingement symptoms due to their proximity to the subacromial space (Figure 6). Patients typically experience pain during overhead activity, mimicking subacromial impingement syndrome. A positive Neer’s or Hawkins-Kennedy may be elicited [58,59]. Notably, the chronic formative phase of this stage may persist for approximately 1 to 6 years, during which calcium deposits gradually and patients are either asymptomatic or experience mild impingement [10]. Consequently, RCCT may be diagnosed unintentionally in this phase during imaging studies for unrelated complaints.

The acute resorptive phase of the calcific stage, however, is responsible for the severe pain seen in RCCT. Lasting from approximately 3 weeks to 6 months, this phase involves an aggressive inflammatory response mounted by macrophages and mononuclear giant cells to remove calcium deposits and restore normal tendon structure [5,10]. This inflammatory reaction is characterized by edema, increased intratendinous pressure, and possible extravasation of calcium crystals into the SASD bursa. Guido et al. reported that patients with sudden-onset unilateral pain at the supraspinatus tendon insertion—often occurring at night—with radiation to the neck, limited range of motion, and rapid symptom progression are likely in the resorptive phase of RCCT [3]. Physical exam findings include tenderness upon palpation of the greater tuberosity, deltoid, bicipital groove, acromial process, and coracoid process, along with local erythema, swelling, muscle atrophy, and increased skin temperature. In some cases, a palpable and painful mass on the anterior surface of the patient’s shoulder may be present as well [60]. All glenohumeral movements including forward flexion, extension, external and internal rotation, abduction, adduction, and overhead activity are described as painful for RCCT patients [10,58,59]. Pain during the midrange of abduction arm between 70 and 120 degrees, known as the painful arc, is observed and reflects impingement of tendons within the subacromial space [58]. These movement restrictions, shoulder weakness, and strength deficits, are attributed to calcium deposits in the supraspinatus, subscapularis, and infraspinatus tendons, which are critical to shoulder motion, mobility, and stabilization.

The post-calcific stage is termed “acute” because it refers to the acute inflammatory symptoms that are characteristic of the resorptive phase it overlaps with [5,10]. The type III collagen that is laid by fibroblasts during remodeling matures into organized type I collagen over 12–16 months [5,10,17,18,28]. It is marked by an improvement or resolution of pain with lingering stiffness or weakness, depending on the severity of RCCT and whether any treatment was sought.

### 4.2 Clinical Manifestations of Rotator Cuff Non-Calcific Tendinopathy

Non-calcific rotator cuff tendinopathy follows a more insidious, chronic course compared to the staged, self-limiting progression of RCCT [13,14]. It results from repetitive microtrauma, mechanical overload, and age-related degenerative changes that compromise tendon structure [13,14]. Unlike RCCT, there is no calcium deposition; instead, the pathology is driven by collagen disorganization, neovascularization, and tenocyte apoptosis that led to gradual tendon weakening.

Patients with non-calcific RCT often report a slow onset of dull, aching shoulder pain that worsens with activity, especially during overhead movements, lifting, or reaching behind the back. In contrast to the sudden, intense pain seen in the resorptive phase in RCCT, symptoms in non-calcific RCT progress gradually and may persist for months to years before patients seek evaluation. On physical exam, pain tends to be localized to the anterolateral shoulder without radiation and is accompanied by tenderness of the greater tuberosity, deltoid insertion, bicipital groove, and acromion process [13,58]. Signs of acute inflammation such as erythema, swelling, muscle atrophy, and warmth are uncommon in non-calcific RCT, as these are related to macrophage phagocytosis of calcium deposits in RCCT. Patients with non-calcific RCT often demonstrate a painful arc due to inflammation, microtears, and subacromial bursitis, leading to positive Neer and Hawkins-Kennedy tests [59]. The functional limitations associated with non-calcific RCT can result in lifelong chronic pain. Without acute pain flares caused by calcific deposits, symptoms of non-calcific RCT are primarily driven by ongoing tendon degeneration and mechanical irritation, which contribute to progressive reductions in shoulder mobility and stability [13,58].

## 5. Ultrasound: Primary Diagnostic Method for Rotator Cuff Tendinopathy

Although the clinical presentations of calcific and non-calcific RCT differ in onset, severity, and inflammation, overlapping symptoms can make clinical diagnosis challenging. Given this variability and the potential for nonspecific physical exam findings, imaging is essential for accurately characterizing the underlying pathology, differentiating between calcified and noncalcified forms, and guiding appropriate treatment strategies.

Ultrasound (US), magnetic resonance imaging (MRI), and magnetic resonance arthrography (MRA) are commonly used imaging modalities for diagnosing rotator cuff tendinopathy. Compared to MRI and MRA, however, US offers several inherent advantages. Current evidence supports the use of MSK ultrasound as the preferential initial imaging modality in the evaluation of rotator cuff disease [60–65]. One major benefit is lower cost, though pricing can vary significantly depending on geographic location and institutional factors [60]. In fact, a study examining the use of diagnostic musculoskeletal (MSK) US versus MSK MRI over a fifteen-year period projected that the use of MSK US could result in $6.9 billion in healthcare savings [60]. These findings highlight the potential for MSK US to serve as a cost-effective alternative to more expensive imaging modalities without compromising diagnostic utility. As healthcare systems increasingly prioritize value-based care, wider adoption of US could lead to substantial economic benefits while maintaining high standards of patient care. In addition to cost, US demonstrates excellent diagnostic accuracy. MRI, MRA, and US are highly effective in assessing rotator cuff tendinopathy. A full-thickness rotator cuff injury can be diagnosed with high accuracy by all three methods: 92.1% sensitivity and 92.9% specificity, 95.4% sensitivity and 98.9% specificity, and 92.3% sensitivity and 94.4% specificity, respectively [66]. Given their comparable accuracy, the combination of lower cost and faster availability of US make it a particularly favorable choice. Accessibility further distinguishes US from other imaging modalities. In a study conducted at a quaternary health center, nearly half of ordered MRIs took longer than 10 days to complete, with a mean wait time of 18.5 days [67]. In contrast, ultrasound equipment is widely distributed—available in 88.56% of metropolitan counties and 74.19% of US counties—making it a far more accessible modality [68]. This availability enhances the ability to extend timely MSK care to underserved and rural areas, where access to advanced imaging like MRIs and MRAs is often limited. Ultrasound also offers rapid turnaround in both image acquisition and interpretation. While MRI results require evaluation and interpretation by a radiologist, followed by communication with a referring physician before informing the patient, MSK US allows for real-time interpretation by a trained clinician performing the scan. This eliminates delays associated with MRI interpretation and reporting. Immediate feedback from MSK US accelerates diagnosis and treatment planning, which can ultimately improve patient outcomes.

This efficiency is especially valuable in evaluating conditions like RCCT, where timely diagnosis can help guide intervention. Calcific tendinopathy involves the deposition of hydroxyapatite calcium crystals in tenocytes and is accompanied by severe pain and stiffness that limits range of motion [65]. The three stages of RCCT lead to various levels of calcium deposition, inflammation, and tissue responses that can be assessed using US. Each stage of RCCT has distinct ultrasound features that correspond with the underlying pathological process and clinical severity. Generally, calcific deposits in the shoulder are visualized on US as hyperechoic artifacts marked by variable levels of acoustic shadow [63]. The pre-calcific stage involves a metaplasia of native tenocytes to chondrocytes, optimizing conditions for calcification to proceed. The formative phase of the calcific stage follows, during which calcium deposits form a chalky-like appearance and appear with clear acoustic borders on US. During the resting phase, hard calcification between tendon fibers leads to focal thickening of the rotator cuff. In the resorptive phase, the appearance of calcific deposits can be altered [69,70]. Macrophages and other immune cells initiate the breakdown of intra-tendinous calcific deposits, leading to an inflammatory response [62]. This results in sonographic patterns that are fragmented, nodular, or cyst-like; however, color Doppler can be used to identify hydroxyapatite crystals due to the proliferation of capillaries and thin-walled vascular channels surrounding them [62,63,69]. While MRI remains more sensitive for detecting labral tears and intra-articular pathology, US reliably detects tendon thickening, hypoechoic degeneration, and neovascularization in a dynamic setting.

Comparatively, non-calcific tendinopathy typically presents with tendon thickening due to a loss of the normal, organized fibrillar pattern. On ultrasound, the tendon initially appears hypoechoic, representing swelling, degeneration, and inflammation. In more advanced cases, the tendon may show focal thinning, or a laminated (disrupted) appearance accompanied by distension of the subacromial-subdeltoid bursa. These findings reflect the underlying chronic degenerative process of tendinosis and tendinopathy, which is characterized histologically by tenocyte rounding, increased ground substance, and failed tendon healing. Color or power Doppler may also reveal detectable neovascularization, a hallmark of chronic tendinopathy, which is typically absent in healthy tendons. The presence of these abnormal vessels—seen in up to 65% of symptomatic shoulders—supports a working diagnosis of rotator cuff tendinopathy, although their presence does not always correlate with pain severity [70–72]. Additionally, dynamic US can reveal functional impingement or tendon instability during active shoulder movement, providing a more complete clinical picture and guiding conservative or surgical decision-making.

## 6. Clinical Decision-Making: Established and Emerging Treatment Strategies

Recently, the priority of conservative methods in treating calcified and non-calcified rotator cuff tendinopathy has increased. The first goal is to control pain and reduce inflammation, which can be accomplished with NSAIDs [73]. To increase muscle strength and improve range of motion, physical therapy and rehabilitation programs are strongly indicated. Exercise-based interventions have also demonstrated significant efficacy in reducing pain and enhancing functional outcomes in patients diagnosed with rotator cuff tendinopathy [74]. While some studies show exercise programs with progressive overload as the most effective physical activity to treat rotator cuff tendinopathies [75,76], there is no consensus on which program is most appropriate as many studies demonstrate contradictory results [77–79]. This may be due to the therapies targeting different structures, such as the rotator cuff muscles or the scapula. While conservative therapies are effective in reducing inflammation and improving functionality in rotator cuff tendinopathy, these treatments may also progress into rotator cuff tears [80]. However, no causality was established, and it is unclear if conservative treatment of rotator cuff tendinopathy is a risk factor for rotator cuff tears.

Interventional approaches for rotator cuff tendinopathy vary depending on whether the condition is calcified or non-calcified. In cases of rotator cuff calcific tendinopathy, commonly employed interventions include ultrasound-guided barbotage, extracorporeal shockwave therapy (ESWT), and corticosteroid injections. Ultrasound-guided barbotage is a minimally invasive technique in which calcium deposits are fragmented and aspirated under real-time ultrasound guidance; this procedure is often followed by a corticosteroid injection to reduce post-procedural inflammation. Ultrasound-guided needling for the treatment of rotator cuff tendinopathy has been demonstrated to be a safe and effective intervention, with a lower relative risk of subsequent surgical intervention compared to corticosteroid injections alone [81,82]. ESWT applies high- or low-energy sound waves to the affected area to fragment calcium deposits and promote tissue regeneration. In rotator cuff tendinopathy, ESWT is particularly effective in reducing pain and improving functioning in the first 6 months [83]. High-energy ESWT may also be more effective than low energy ESWT but could be associated with increased risk of pain and hematomas during and immediately following the procedure [84]. Corticosteroid injections are also used to treat both calcified and non-calcified rotator cuff tendinopathies and are most effective in short-term pain relief. Compared to oral NSAIDs, corticosteroid injections have demonstrated superior increases in shoulder range of motion and pain relief after 3 months [85]. However, evidence supporting sustained pain relief beyond eight weeks is limited, and rotator cuff corticosteroid injections have been associated with potential adverse effects, such as discomfort, cost, and potential to accelerate tendon degeneration [86]. Given the range of interventional options, treatment selection should be individualized based on the type of tendinopathy, symptom duration, and risk-benefit profile of each modality.

**Figure 7:**
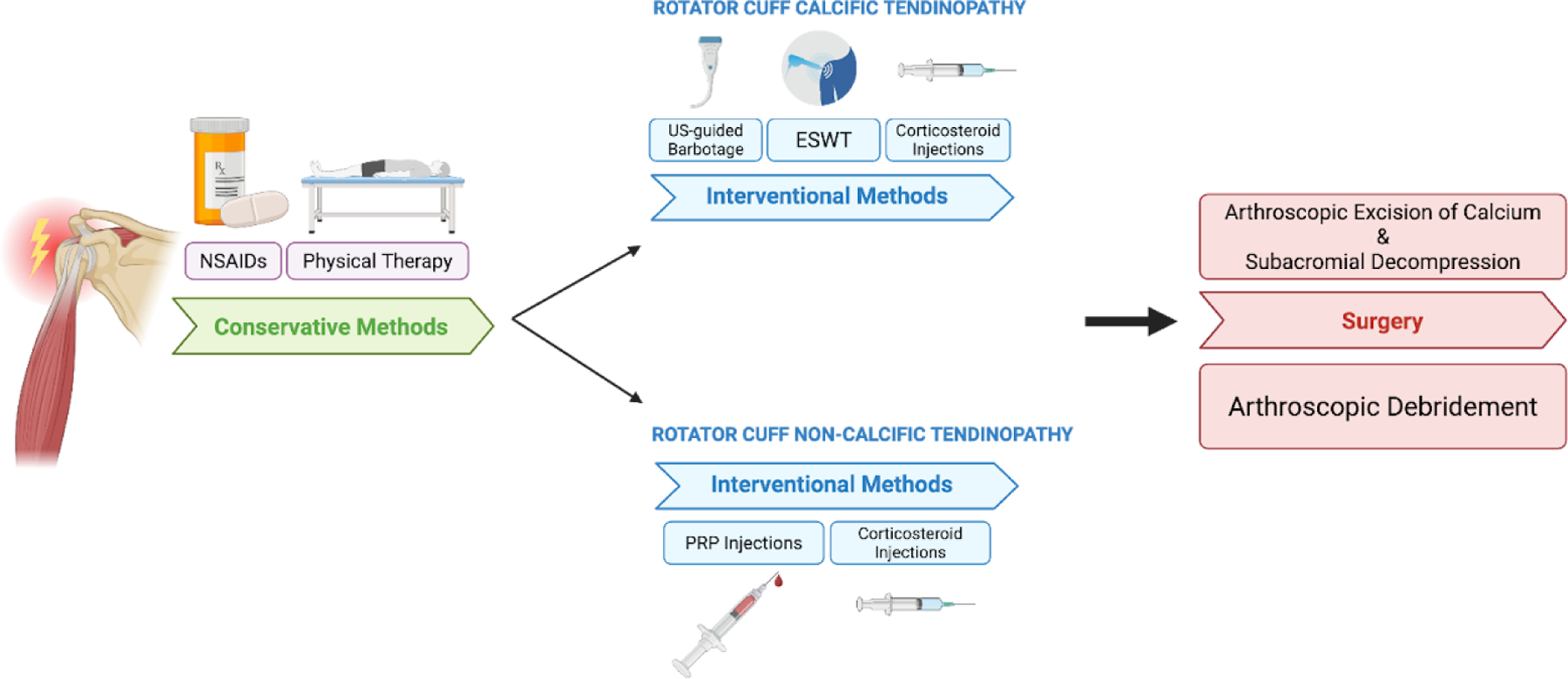
Stepwise treatment algorithm for rotator cuff tendinopathy. Management begins with conservative approaches such as NSAIDs and physical therapy. For patients with persistent symptoms, treatment diverges based on the presence of calcific versus non-calcific pathology. Surgical intervention is reserved for refractory cases—arthroscopic excision of calcium with subacromial decompression for RCCT, and arthroscopic debridement for non-calcific cases.

Non-calcified rotator cuff tendinopathies are treated interventionally, primarily with corticosteroid injections and platelet-rich plasma (PRP) injections. PRP rotator cuff injections have demonstrated mixed results, as some studies claim the injections reduce rates of surgery and reduce pain in non-calcific rotator cuff tendinopathy [87], while others suggest no significant functional differences in the shoulder as compared to a placebo [88]. The available evidence on PRP injections may be conflicting due to variability in settings, indications, and clinical outcomes. Therefore, further well-designed, standardized clinical studies are necessary before PRP injections can be recommended as an evidence-based treatment for non-calcified rotator cuff tendinopathy. Other emerging treatment modalities for rotator cuff injuries include prolotherapy and percutaneous tenotomy. Prolotherapy involves the injection of a dextrose-based irritant solution to provoke an inflammatory response and promote tissue repair [89], while percutaneous tenotomy employs mechanical disruption of degenerative tendon tissue to stimulate a regenerative healing process [90]. Although these interventions show promise, further high-quality, randomized controlled trials are needed to establish their clinical efficacy and long-term outcomes (Figure 7).

## 7. Prognosis and Outcomes

Rotator cuff tendinopathy has a generally favorable prognosis, particularly with early diagnosis and appropriate treatment. Conservative approaches for both non-calcific RCT and RCCT, including exercise-based physical therapy and NSAIDs, have demonstrated success rates of 60–80% in improving pain and function over a period of several months [7]. Calcific tendinopathy may resolve spontaneously in some cases due to natural resorption of calcium deposits—which is why conservative management is preferred—but persistent or severe cases can result in prolonged discomfort and limited mobility [91]. Factors that indicate failed conservative treatment in RCCT include large, bilateral deposits within the tendon structure known as the “calcific bulging sign” and spread of the calcification to locations such as the anterior edge of the acromion [9]. Conservative treatment is typically continued as a first-line approach, but there is currently no defined timeframe for determining when it has been fully exhausted, and invasive options should be considered. The patient’s pain tolerance and the degree of disability are key criteria for transitioning to interventional therapy.

In cases of symptoms from non-calcific RCT that fail to respond to conservative treatments, arthroscopic debridement offers a minimally invasive option to remove degenerated tendon tissue, reduce inflammation, and restore shoulder function. This approach has been shown to substantially improve pain and range of motion in patients with chronic tendinopathy without full-thickness tears. In a study by Budoff et al., 77% of the 60 patients who underwent this procedure reported minimal to no pain at a 114-month follow-up [92]. As a tissue-preserving alternative that reduces local inflammation, arthroscopic debridement points to promising outcomes and may be appropriate for patients who are not candidates for more extensive surgical interventions.

Patients with symptomatic calcific tendinitis who do not achieve satisfactory relief after at least six months of non-surgical management often seek further evaluation by a physician [7]. Surgical intervention is typically considered when large calcific deposits cause painful mechanical impingement. Long-term outcomes are influenced by several factors such as patient age, size and density of calcific deposits, symptom duration, degree of tendon degeneration, and treatment adherence [7]. For instance, younger patients tend to heal more quickly due to greater tendon cell regenerative capacity. Dense, well-defined deposits are associated with better postoperative outcomes, as they are easier to localize and remove than smaller or fragmented calcific deposits. Similarly, patients with shorter symptom duration often exhibit less tendon degeneration and inflammation overall, leading to enhanced recovery relative to those with prolonged symptoms or coexisting shoulder pathologies. Most patients who undergo surgical management for RCCT also have reported significant reductions in shoulder pain and improved functional outcomes compared to those treated nonoperatively [93,94].

## 8. Conclusion

Rotator cuff tendinopathy, encompassing both calcific and non-calcific subtypes, is a prevalent cause of shoulder dysfunction with distinct pathophysiological mechanisms, clinical progression, and treatment considerations. Rotator cuff calcific tendinopathy is characterized by a staged process of calcium hydroxyapatite deposition and eventual resorption, driven by the metaplastic transformation of tenocytes into chondrocyte-like cells and the activation of mineralization pathways. In contrast, non-calcific RCT reflects a chronic degenerative process including collagen disorganization, tenocyte apoptosis, and matrix degradation. These pathological changes are precipitated by intrinsic aging and extrinsic mechanical factors. Such differences in these two classifications of rotator cuff tendinopathy underscore the need for precise diagnostic strategies and individualized therapeutic approaches.

Musculoskeletal ultrasound (MSK US) has become an essential tool in evaluating RCT, offering dynamic, real-time imaging, cost-efficiency, and diagnostic accuracy comparable to MRI. Not only does ultrasound differentiate calcific from non-calcific rotator cuff tendinopathies, but it also characterizes deposit morphology, vascularity, and associated bursal or tendon pathology. The accessibility and immediacy of US interpretation further enhances its role in timely clinical decision making, especially in primary care and resource-limited communities.

Conservative treatment remains the first-line approach for both subtypes of RCT, with NSAIDs and exercise-based based physical therapy demonstrating 60–80% success in improving pain and function over several months. RCCT may spontaneously resolve during the post-calcific remodeling phase, but large, bilateral, or anteriorly located deposits may result in persistent mechanical impingement. For these patients with painful symptoms, interventional options like ultrasound-guided barbotage, ESWT, or surgery may be considered. In non-calcific RCT, chronic tendon degeneration may require additional interventions. While corticosteroid injections offer short-term relief, minimally invasive options like platelet-rich plasma (PRP) therapy and arthroscopic debridement have shown potential for sustained improvement by addressing underlying tendon pathology.

Despite advancements in diagnosis and treatment, several areas warrant further research. Well-designed, standardized clinical trials could help evaluate long-term efficacy of emerging therapies like PRP, prolotherapy, and percutaneous tenotomy. In addition, greater insight into the molecular drivers of tendon mineralization in RCCT and the degenerative cascade in non-calcific RCT will be critical for guiding targeted, mechanism-based interventions. Ultimately, this review aims to enhance understanding of the divergent mechanisms and overlapping clinical features of RCCT and non-calcific RCT to support more personalized and effective care for patients with rotator cuff tendinopathy.

## 9. Key Points

- Rotator cuff tendinopathy includes non-calcific and calcific subtypes, with overlapping clinical features but distinct pathophysiologic mechanisms and treatment implications.
- A combination of both intrinsic and extrinsic factors—like natural aging, collagen degeneration, genetic predisposition, poor vascularity, acromial shape, scapular kinetics, and overhead activity—contribute to rotator cuff tendinopathies.
- Rotator cuff calcific tendinopathy involves calcium hydroxyapatite deposition driven by the metaplasia of tenocytes to chondrocytes and poor vascularization, whereas non-calcific RCT is attributed to collagen matrix breakdown and chronic overload.
- Rotator cuff calcific tendinopathy presents with a sudden, severe pain during the resorptive phase that is usually self-limiting, while non-calcific RCT manifests as gradual, activity-related pain and stiffness.
- Ultrasound is considered a primary diagnostic tool that differentiates echogenic deposits with acoustic shadowing in RCCT versus tendon thickening, hypoechogenicity, and neovascularity in non-calcific RCT.
- The first-line treatment for rotator cuff tendinopathies is conservative, emphasizing rest, NSAIDs, physical therapy, and lifestyle modification, followed by interventional and surgical considerations if symptoms persist.
- If conservative treatment fails, persistent rotator cuff calcific tendinopathy may require ultrasound-guided barbotage, ESWT, or arthroscopic excision of calcific deposits, while chronic non-calcific RCT may respond to PRP injections or arthroscopic debridement.
- Prognosis of non-calcific and calcific rotator cuff tendinopathy is dependent on early identification and management of modifiable intrinsic and extrinsic factors because delayed intervention increases the likelihood of irreversible tendon degeneration and the need for surgical repair.
